# Cybersecurity on a budget: Evaluating security and performance of open-source SIEM solutions for SMEs

**DOI:** 10.1371/journal.pone.0301183

**Published:** 2024-03-28

**Authors:** Jawad Manzoor, Abdul Waleed, Abdul Fareed Jamali, Ammar Masood

**Affiliations:** 1 School of Computer Science, University of Galway, Galway, Ireland; 2 National Center for Cyber Security, Islamabad, Pakistan; 3 Faculty of Computing and AI, Air University, Islamabad, Pakistan; Institute of Theoretical and Applied Informatics Polish Academy of Sciences: Instytut Informatyki Teoretycznej i Stosowanej Polskiej Akademii Nauk, UKRAINE

## Abstract

The proliferation of cyber threats necessitates robust security measures to safeguard critical assets and data in today’s evolving digital landscape. Small and Medium Enterprises (SMEs), which are the backbone of the global economy are particularly vulnerable to these threats due to inadequate protection for critical and sensitive information, budgetary constraints, and lack of cybersecurity expertise and personnel. Security Information and Event Management (SIEM) systems have emerged as pivotal tools for monitoring, detecting, and responding to security incidents. While proprietary SIEM solutions have historically dominated the market, open-source SIEM systems have gained prominence for their accessibility and cost-effectiveness for SMEs. This article presents a comprehensive study focusing on the evaluation of open-source SIEM systems. The research investigates the capabilities of these open-source solutions in addressing modern security challenges and compliance with regulatory requirements. Performance aspects are explored through empirical testing in simulated enterprise-grade SME network environments to assess resource utilization, and real-time data processing capabilities. By providing a rigorous assessment of the security and performance features of open-source SIEM systems, this research offers valuable insights to cybersecurity practitioners, organizations seeking cost-effective security solutions, and the broader academic community. The findings shed light on the strengths and limitations of these systems, aiding decision-makers in selecting the most suitable SIEM solution for their specific requirements while enhancing the cybersecurity posture of SMEs.

## 1 Introduction

SMEs play a crucial role in driving innovation, yet they fail to adequately strategize their cybersecurity defense. One of the reasons for this oversight is underestimating the risks and impact of cyber attacks. There is often a misconception that cybercriminals only go after large, high-profile organizations. Unfortunately, this couldn’t be further from the truth. Verizon’s data breach investigation report [1] reveals that nearly 43% of cyber attacks are targeted at SMEs. Another reason for the inadequate cybersecurity posture of SMEs is that due to limited financial and human resources, they struggling to keep up with the constant advancements in this rapidly evolving domain. SMEs often find themselves unprepared to select the right tools to safeguard their assets, thereby jeopardizing their business continuity. Since the outbreak of the COVID-19 pandemic and the lockdowns that ensued worldwide, organizations have adopted remote work and the employees access the organization’s systems remotely from their homes. This has created new opportunities for malicious actors and an increase in cyber attacks has been observed following the COVID-19 pandemic. The European Union Agency for Cybersecurity, ENISA [2], found that cybersecurity challenges were exasperated further by the impact of the COVID-19 pandemic and that SMEs were unprepared to cope with these challenges. A recent survey of 85 UK-based SMEs explored their threat and coping appraisals toward cyber attacks. A major concern shown by SMEs was keeping mobile devices safe and avoiding phishing attacks [3]. Employing the defense-in-depth strategy, organizations deploy a number of security solutions such as Next Generation Intrusion Detection and Prevention Systems (NG-IDPS), firewalls, antivirus solutions, network segmentation etc. These security solutions are deployed across complete network infrastructure to ensure real-time security through continuous monitoring and response [4].

Despite the availability of a variety of security solutions, analysts have a hard time monitoring multiple dashboards simultaneously and correlating events from different security devices. Moreover, these devices generate a huge amount of data (logs) in multiple formats thus overwhelming the log management. This issue is particularly important for SMEs that usually have limited human resources and managing security can often be a part-time job for a single individual [5]. This makes them an easy target for cybercriminals. Therefore, a pragmatic approach for SMEs is unified security management. SIEM system facilitates this with efficient collection of data from disparate log sources into a single system for real-time analysis [6] delivered to a single console. SIEM system itself is not an active monitoring device in the network but is a powerful security solution to monitor logs from multiple devices and correlate them in real-time to observe any malicious activity that may be overlooked by other network parametric defense solutions [7].

There are two broad categories of SIEM solutions, commercial and open-source, with inherent benefits and limitations. Commercial SIEM solutions are quite mature and provide full enterprise-level coverage, albeit with huge licensing costs. For instance, the three-year total cost of ownership (TCO) for LogRhythm and SolarWinds LEM starts from $50,000 and the same for AlienVault USM, IBM Qradar and HP ArchSight starts from $250,000. Open-source solutions do not incur any cost and are open for modification or customization; however, they are usually restricted in terms of features and lack customer support. There are a number of open-source SIEM solutions available in the market, however, the selection of an optimum SIEM solution can be a difficult job for most SMEs due to the lack of expertise and resources to perform detailed comparisons and testing of security and performance features of each. While evaluations of commercial SIEM solutions are often provided by their vendors, and institutes such as Gartner [8], InfoTech Research Group, TechTarget, InfoWorld and CSO Online also perform a comparative analysis of commercial SIEM solutions annually, such evaluations and comparisons are largely missing for open-source SIEM solutions. The innovation, security analysis and performance evaluation of open-source SIEM solutions rely on the research community. Researchers are actively working to improve these solutions by integrating open-source intelligence (OSINT) and Artificial Intelligence (AI).

This research work aims to investigate the technical underpinnings of SIEM and perform an experimental evaluation of the security features and performance of state-of-the-art open-source SIEM solutions that suit the security and compliance needs of SMEs while staying within the resource constraints that are inherent in smaller organizations. The main research questions that we want to answer are the following:

- What are the key security features and capabilities offered by open-source SIEM solutions, and how do they compare to commercial SIEM solutions?
- What are the performance benchmarks of different open-source SIEM solutions under high traffic load and event data volume?
- What are the scalability limitations of open-source SIEM solutions, and how do they perform as the size and complexity of the monitored network infrastructure grow?
- How do open-source SIEM solutions address compliance and regulatory requirements, and what kind of reports do they provide for auditing purposes?
- To what extent do open-source SIEM solutions support cyber threat intelligence integration to identify emerging threats?

Prior work on the evaluation of open-source SIEM systems largely focused on theoretical analysis of architectural components and basic features; thus, omitting important performance metrics such as Events Per Second (EPS) assessment and evaluation of various security features. Secondly, SIEM is a constantly evolving field with new entrants coming to market and new innovative features being added to existing products. Therefore, prior studies can become outdated, and new research on the security and performance evaluation of state-of-the-art open-source SIEM solutions is necessary. To this end, we provide a detailed security and performance evaluation of the latest open-source SIEM solutions. Our main contributions are:

- We have performed a detailed security and performance evaluation of the most popular and widely used open-source SIEM solutions.
- We have developed a ranking mechanism based on essential and desirable features of SIEM.
- We have deployed an enterprise-grade test bed to simulate a real-world network of SMEs to evaluate SIEM solutions in an operational environment. In particular, the test network was used to establish the veracity of EPS claims of SIEM solutions.
- We have identified several shortcomings in current open-source SIEM solutions and discussed potential enhancements that will be implemented in our future work.

The rest of the paper is organized as follows: Section 2 presents SIEM architecture. Section 3 summarizes the previous work on the evaluation of SIEM solutions. Section 4 presents a brief overview of open-source SIEM solutions. Section 5 describes our evaluation strategy, parameters and the testing environment. We discuss the results in detail in Section 6, and Section 7 concludes the paper and presents some future research directions.

## 2 SIEM architecture

**Fig 1:**
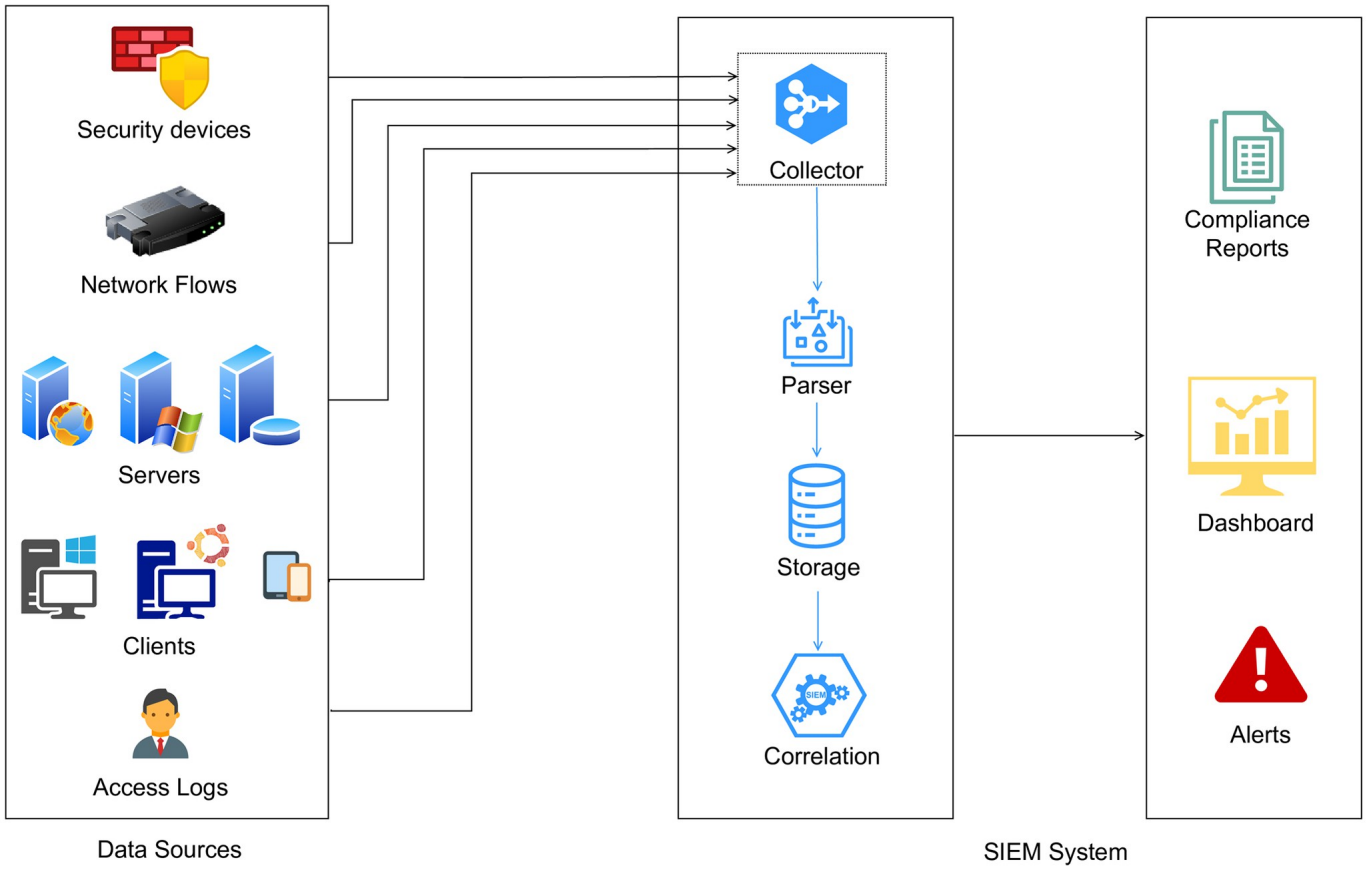
SIEM architecture.

SIEM system is a comprehensive cybersecurity solution that provides real-time monitoring and analysis of an organization’s security posture. It helps in detecting and responding to security incidents by collecting event data from various sources and correlating them. The architecture of a SIEM system as shown in Fig 1 comprises the following major components:

### 2.1 Data sources

The data collected from a large number of sources across an organization’s network and infrastructure is the foundation of a SIEM system. Some common data sources include Firewalls, Intrusion Detection and Prevention Systems (IDPS), Intrusion Prevention Systems (IPS), antivirus software, logs from operating systems, databases, web servers and cloud-based applications and services, data from individual workstations, laptops, and mobile devices, and user authentication and access logs. Network flow data is also an important source. External data sources can also provide information about known threats and vulnerabilities in the form of threat intelligence feeds.

### 2.2 Data collection and normalization

Data from a wide range of sources is collected by a SIEM system using agents, APIs, or log forwarding mechanisms. Since each data source has its own format, the collected data goes through a normalization and parsing process. SIEM solutions usually provide built-in parsers for common data sources. Some SIEM solutions also allow you to write custom parsers for new data sources. Normalization ensures that all data is converted into a common format for analysis.

### 2.3 Data storage

The normalized data is then stored in a centralized repository. Some SIEM solutions use relational database management systems (RDBMS) such as MySQL, PostgreSQL, or Microsoft SQL Server to store structured event data. Others leverage NoSQL databases like MongoDB or Elasticsearch to handle unstructured or semi-structured log data. The centralized storage allows for efficient querying, analysis, and reporting. Large volumes of historical security data are usually stored using data warehousing solutions like Amazon Redshift or Snowflake.

### 2.4 Correlation engine

The correlation engine analyzes the collected data to identify patterns and potential security incidents by correlating events from multiple sources. There are different methods of correlation analysis such as statistical method, rule based reasoning, case-based reasoning, model-based reasoning, bayesian reasoning, and graph-based method.

### 2.5 Dashboard, reporting and alerting

SIEM systems provide a dashboard to monitor the organization’s security posture in real time. Dashboards often include visualization of the data in the form of charts, graphs, and widgets. Reports can be generated regularly or on demand for compliance, incident analysis, and executive summaries. The dashboard also shows alerts generated by the correlation engine when a security incident or suspicious activity is identified. Alerts can trigger notifications, such as emails, SMS messages, or integrations with incident response systems. Alerts are usually categorized by severity to prioritize incident response efforts.

## 3 Related work

The literature extensively discusses the design of SIEM systems, their applicability to new domains, feature improvement and evaluation of various SIEM solutions available in the market.

Prior studies related to the evaluation of SIEM solutions are more focused on the theoretical comparison of architectural modules and basic capabilities of these systems and the evaluation criteria have been very subjective. To the best of our knowledge, a comprehensive evaluation of the security and performance of popular open-source SIEM solutions specifically targeted at SMEs is largely missing.

### 3.1 Research on improving SIEM

Sornalakshmi [9] developed methods to detect zero-day attacks by monitoring the modification in specific system parameters. They also develop methods to detect DoS attacks by monitoring the web server logs.

Bryant et al. [10] propose a hybrid kill-chain framework as a novel configuration of SIEM. They develop a new log ontology to normalize sensor data and create new SIEM correlation rules. They tested their methodology against a baseline configuration and the results showed improvement in detection rates and reduction in the number of false positive alerts.

Menges et al. [11] proposed an architecture of SIEM that meets the privacy requirements of the General Data Protection Regulation (GDPR). Detken et al. [12] proposed a security system, which extends existing Network Access Control (NAC) systems with SIEM functionality, additional analyzing methods and dynamical compliance support. One of the aims of the proposed security platform is to make it affordable for SMEs in contrast to other commercial SIEM systems.

Bhatt et al. [13] discussed the operational challenges of using SIEM systems including rule creation and management, lack of contextual information, ad hoc use of long-term data. They also highlight future big data challenges including event collection, storage, correlation and visualization. However, no technical solutions are provided and these problem areas are left open for future research.

### 3.2 SIEM for critical infrastructures

Security in mainstream computer networks is different from security in critical infrastructure networks, due to their unique nature and requirements. The number of attacks against critical infrastructure has significantly increased. One example is the attack on SCADA system using the Stuxnet malware [14]. SCADA attack by the “Sandworm” hacker group on the Ukrainian power grid attack is another example [15]. Several research studies have investigated the suitability of SIEM solutions in critical infrastructures. Hindy et al. [16] provide a new SIEM methodology that detects anomalies in a SCADA-controlled water system. They group the attacks into 14 categories and conduct an evaluation of SIEM performances through operational scenarios. Cinque et al. [17] study SIEM in the context of the air traffic control domain which is a mission-critical system. They discuss the challenges in addressing the massive volumes of highly unstructured text logs that are generated by these systems and their integration with SIEM systems. The energy industry is another sector that is a common target of cyber attacks. According to the IBM Security X-Force threat intelligence index [18], the attacks on the energy sector have increased by 23% in 2022 as compared to the previous year. The use of SIEM systems to protect the energy industry against cyber attacks is an active research area. Cerullo et al. [19] evaluated the benefits of SIEM solutions in detecting various attacks including sleep deprivation, GPS spoofing and DDoS on power grid infrastructures. Gonzalez et al. [20] provided a survey of the most widely used SIEMs in critical infrastructures. Kotenko et al. [21] proposed an Attack Modeling and Security Evaluation Component (AMSEC) as a detection engine in SIEM for industrial 4.0 devices, based on the National Vulnerability Database (NVD) security repository, attack graphs and known attacks. A detailed system architecture and prototype were also described by the authors. Moreover, a comparison with other vulnerability analysis tools such as OpenSKE, COMNET III, Securl Tree, Nessus, and Symantec ESM is performed. This work is limited to the evaluation of targeted vulnerability analysis tools and as such doesn’t consider the complete SIEM systems.

### 3.3 SIEM for IoT networks

Casola et al. [22] presented a monitoring tool for IoT systems based on the extension of the Montimage network monitoring tools. Choi [23] proposed a scheme to minimize security vulnerabilities and threats in IoT devices to improve the security of the IoT service environment. Mármol et al. [24], proposed a SIEM system for IoT using Ethereum blockchain named as BSIEM; where the security of logs and event files is ensured using smart contracts. While the authors also claimed security evaluation of the proposed system; yet, the assurance remains focused on the log management system. The work was extended as BlockSIEM solution for smart cities [25] and the system was evaluated under different use case scenarios. However, very limited performance evaluation was carried out thus missing basic features such as EPS evaluation. Moreover, no security evaluation was conducted. Leszczyna et al. [26], proposed criteria for the evaluation of AlienVault OSSIM, Prelude [27] and Cyberoam iView [28] in a smart grid environment. The evaluation criteria are based on the basic SIEM features and non-functional requirements for SIEM deployment.

### 3.4 Research on SIEM evaluation

Sekharan et al. [29] evaluated commercial SIEM solutions (IBM QRadar [30], HP ArcSight [31], Splunk [32], and LogRhythm [33]) against the architectural components and basic SIEM capabilities. They did not evaluate any open-source solution.

Safarzadeh et al. [34] presented qualitative criteria for the evaluation of capabilities and architectural components of SIEM solutions, where each component and capability is scored based on its effectiveness. ArcSight ESM commercial SIEM solution, AlienVault OSSIM open-source SIEM solution and E-SIEM self-developed SIEM solution were evaluated using this criteria. The paper lacks coverage of other popular open-source SIEM systems such as Wazuh and SIEMonster. Moreover, it does not consider a parametric security evaluation of the targeted solutions.

Christopher et al. [35] performed the analysis of architectural components and basic SIEM capabilities of open-source SIEM solutions, OSSIM, Prelude and Log management solutions (ELK stack). Kavčič et al. [36] analyzed the basic SIEM capabilities of IBM QRadar, Splunk, SIEMonster and OSSIM. Sepúlveda et al. [37] discussed the basic functionalities of Splunk ES, Prelude, Wazuh, and OSSIM. However, these studies do not consider evaluation with respect to performance and security features.

Other researchers opted to evaluate the capabilities of one specific SIEM solution. Mulyadi et al. [38] deployed and evaluated the effectiveness of Elastic Stack. Thiele et al. [39] deployed and analyzed the basic capabilities of the community version of SIEMonster. Särkisaari et al. [40], analyzed basic SIEM capabilities and deployed Wazuh in a test environment. Bernardo et al. [41] evaluated the attack detection capability of Wazuh in a test environment by simulating multiple attacks. However, other open-source SIEM solutions such as OSSIM and SIEMonster were dropped due to insufficient documentation and lack of resources required to deploy the solution. Hence these studies did not provide a comparative analysis with other SIEM solutions.

Nabil at al. [42] perform a theoretical comparison of OSSIM, ELK and LogPoint SIEM solutions by considering log collection, normalization, correlation and reporting. They also perform a small integration test by configuring CheckPoint firewall and OSSEC HIDS with ELK. The reason for the selection of this particular combination is not clear.

Thakur et al. [43] give an analysis of HP ArcSight by discussing its features such as event analysis, correlation engine, policies and reporting. They also discuss case studies of some attacks that can be detected by AlientVault USM, including SQL Injection, Watering Hole Attack and Malware infection. The authors do not delve into any technical details and do not provide any experimental evaluation.

### 3.5 Institutional reports on SIEM evaluation

Research into security products by various institutes mostly comprises commercial considerations. Various institutions conduct evaluations of security solutions and report their findings regularly. Gartner [8], for example, conducts evaluations of commercial SIEM and releases annual reports that position SIEM vendors within categories such as market leaders, challengers, niche players, or visionaries in their well-known magic quadrant. Another institute Info-Tech [44, 45] publishes technical reports on the SIEM vendor landscape, comparing the pros and cons of major players. Similarly, TechTarget [46] also publishes reports that compare SIEM solutions based on their performance, sales execution/pricing and customer experience. These reports are designed to assist SIEM buyers in selecting the most suitable solution for their businesses. However, a shortcoming of these reports is that they only cover commercial SIEM solutions that are not financially viable for many SMEs.

### 3.6 Other related work

Pavlik et al. [47] critically assessed SIEM deployments in various cloud environments including Infrastructure as a Service (IaaS), Platform as a Service (PaaS) and Software as a Service (SaaS) model. They selected one open-spurce product AlientVault OSSIM and one commercial product IBM Qradar for this analysis. They conclude that while it is possible to deploy OSSIM using IaaS, there are a number of limiting factors. Deploying OSSIM using PaaS and SaaS is not practical. On the other hand, Qradar is very suitable for IaaS, less so for PaaS and SaaS. Mokalled et al. [48] presented customer-driven criteria for the selection of SIEM for an enterprise. The proposed criteria are expected to help consumers in considering all the basic and advanced factors for evaluating a SIEM solution for an enterprise. A detailed model based on performance factors and features of the SIEM system is also examined. They also proposed an approach [49] to support companies that are seeking to adopt SIEM systems into their environments. They provide a set of suitable technological and business requirements that are believed to be valuable in a SIEM system. They provide a template with 14 features: (1) level of compliance, (2) complexity, (3) capability, (4) robustness, (5) scalability, (6) vision, (7) installation duration, (8) licensing, (9) support, (10) training, (11) additional features, (12) integration with third parties, (13) vendor skills, (14) price. This template must be completed for each candidate SIEM solution by assigning appropriate weight/importance to each feature. However, the actual evaluation of a SIEM system using the proposed criteria is not performed. A drawback of the proposed approach is that it is subjective and most SMEs lack the human resources with appropriate technical skills to correctly evaluate each of these features. Secondly, some of the features like complexity, capability, vision, etc. are too vague. Finally, this is not a practical approach because deploying and testing a large number of SIEM solutions is a very time-consuming and error-prone process.

Based on the consolidated comparison of discussed works, as indicated in Table 1, the following conclusions can be drawn:

- Some prior studies studied SIEM solutions in specialized domains such as IoT, smart grids, industrial control systems, etc.
- Some studies considered only commercial SIEM solutions for evaluation, which are suitable only for large enterprises due to huge licensing costs.
- Another group of research studies provided theoretical comparisons of open-source SIEM solutions but the experimental evaluation of their performance and security features is not performed.
- Others have performed experimental evaluation, but only for a single SIEM solution in isolation, which does not provide a head-to-head comparison of various SIEMs.

Our objective is to fill in this research gap by providing a comprehensive analysis of the latest open-source SIEM solutions, considering both performance and security features particularly targeted at SMEs. We propose a ranking mechanism for SIEM systems based on essential and desirable features. The outcome will aid SMEs in effective decision-making.

**Table 1:** Comparison of related work.

Ref	Wazuh	OSSIM	SIEMonster	Elastic Security	A1	A2	A3
Safarzadeh et al. [34]	*χ*	✓	*χ*	*χ*	✓	*χ*	*χ*
Christopher et al. [35]	*χ*	✓	*χ*	✓	*χ*	*χ*	*χ*
Nabil et al. [42]	*χ*	✓	*χ*	✓	*χ*	*χ*	*χ*
Kavčič et al. [36]	*χ*	✓	✓	*χ*	*χ*	*χ*	*χ*
Sepúlveda et al. [37]	✓	✓	*χ*	*χ*	*χ*	*χ*	*χ*
Mulyadi et al. [38]	*χ*	*χ*	*χ*	✓	*χ*	*χ*	*χ*
Thiele et al. [39]	*χ*	*χ*	✓	*χ*	*χ*	*χ*	*χ*
Särkisaari et al. [40]	✓	*χ*	*χ*	*χ*	*χ*	*χ*	*χ*
Bernardo et al. [41]	✓	*χ*	*χ*	*χ*	*χ*	*χ*	*χ*
Leszczyna et al. [26]	*χ*	✓	*χ*	*χ*	✓	*χ*	*χ*
Our Work	✓	✓	✓	✓	✓	✓	✓

***Abbreviations***:

A1 Evaluation Criteria A2 Performance Evaluation A3 Security Feature Evaluation

## 4 Open-source SIEM solutions

Several companies including IBM, Microsoft, LogRhythm, Securonix, and Exabeam have developed powerful SIEM products, however, these products are not affordable for most SMEs due to their huge licensing costs. There are also several open-source SIEM solutions with different features and capabilities to detect attacks and anomalies in an organization’s infrastructure. Open-source and free solutions are viable options for SMEs. Nonetheless, selecting the best solution from all the available options in the market can be a challenging task for SMEs as it requires an in-depth understanding of security features, performance parameters and a specialized knowledge base. To this end, we perform a comprehensive security and performance evaluation of the latest and most widely used open-source or free SIEM systems namely, Wazuh, SIEMonster, OSSIM, Elastic Security, Splunk, and Apache Metron. These solutions have been selected after careful consideration of various features and capabilities they offer to meet different security monitoring and analysis needs, providing SMEs with options for implementing effective threat detection and response strategies. A brief review of each SIEM solution is presented next before discussing our evaluation methodology in detail in Section 5.

### 4.1 Wazuh

Wazuh [50] is an open-source network security monitoring solution. It evolved from OSSEC, which itself is an open-source host-based intrusion detection system (IDS). Wazuh provides a wide range of plugins, that can be integrated to enhance monitoring for multiple security devices. It also provides secure log collection, vulnerability scanning, user-based access control and authentication.

### 4.2 SIEMonster

SIEMonster combines the properties of multiple open-source security solutions and provides a unified single platform that leverages secure log collection, user authentication, and access control. SIEMonster’s architecture utilizes the Wazuh agent for endpoint monitoring, ELK Stack for data collection, processing, storage and visualization, RabbitMQ for queuing and SearchGuard for encryption and authentication. The community edition of SIEMonster is free whereas the professional, enterprise and MSSP editions require a subscription ranging from $600 to $5000 per month.

### 4.3 AlienVault OSSIM

Open-Source Security Information Management (OSSIM) [51] is an open-source variant of the commercially available AlienVault Unified Security Management (USM). OSSIM provides all the basic SIEM functionalities along with vulnerability scanning and asset discovery. The downside is that it is limited in terms of event processing speed and built-in correlation rules and lacks a threat intelligence module. OSSIM combines several open-source projects including Munin, Nagios, NFDump, OpenVAS, Snort, Suricata and TCPTrack.

### 4.4 Elastic security

Elastic Security previously known as Elastic SIEM [52] is also a freely available solution for users. It is built on the Elastic Stack and is capable of detecting host and network-based complex security events using pre-built rules. Other features include vulnerability scanning, user authentication, and access control. The standard version of Elastic Security is free whereas Gold, Platinum and Enterprise versions are paid.

### 4.5 Splunk

Splunk Enterprise is an efficient data management platform that provides analysis and visualization of data [53]. Splunk Enterprise Security (SES) is another solution, which is capable of correlating events for security analysis and identifying malicious and anomalous events. The free version of Splunk Enterprise known as SplunkFree is restricted to storing only up to 500 MB of data and does not contain advanced features that are available in SES. Since SplunkFree does not satisfy the requirements of SMEs, we have not included it for detailed evaluation.

### 4.6 Apache Metron

Apache Metron is a security analytic framework that has evolved from the Cisco OpenSOC project [54]. It provides network security monitoring capability for deeper insights into network activities. However, the project has reached its end of life and is no longer supported. Hence, Apache Metron has not been included in the further detailed evaluation.

Based on this initial review, the four products; Wazuh, SIEMonster, OSSIM, and Elastic Security are shortlisted for detailed evaluation.

## 5 Evaluation and results

In order to ensure a meaningful comparison between the shortlisted SIEM solutions we undertook their quantitative evaluation instead of a simple subjective assessment. It is based on experimental measurements that yield unbiased results. We have designed a scoring mechanism that evaluates the SIEM solutions on the basis of their level of implementation of a set of important features. The scoring mechanism is developed by keeping in view the requirements of SMEs. Our evaluation methodology comprises 1) Performance evaluation and 2) Feature evaluation.

### 5.1 Performance evaluation

The performance of a SIEM system is normally measured in terms of EPS, which is the number of events that can be processed per second. These events come from a diverse range of devices and the SIEM system parses and correlates these events according to predefined rules.

**Fig 2:**
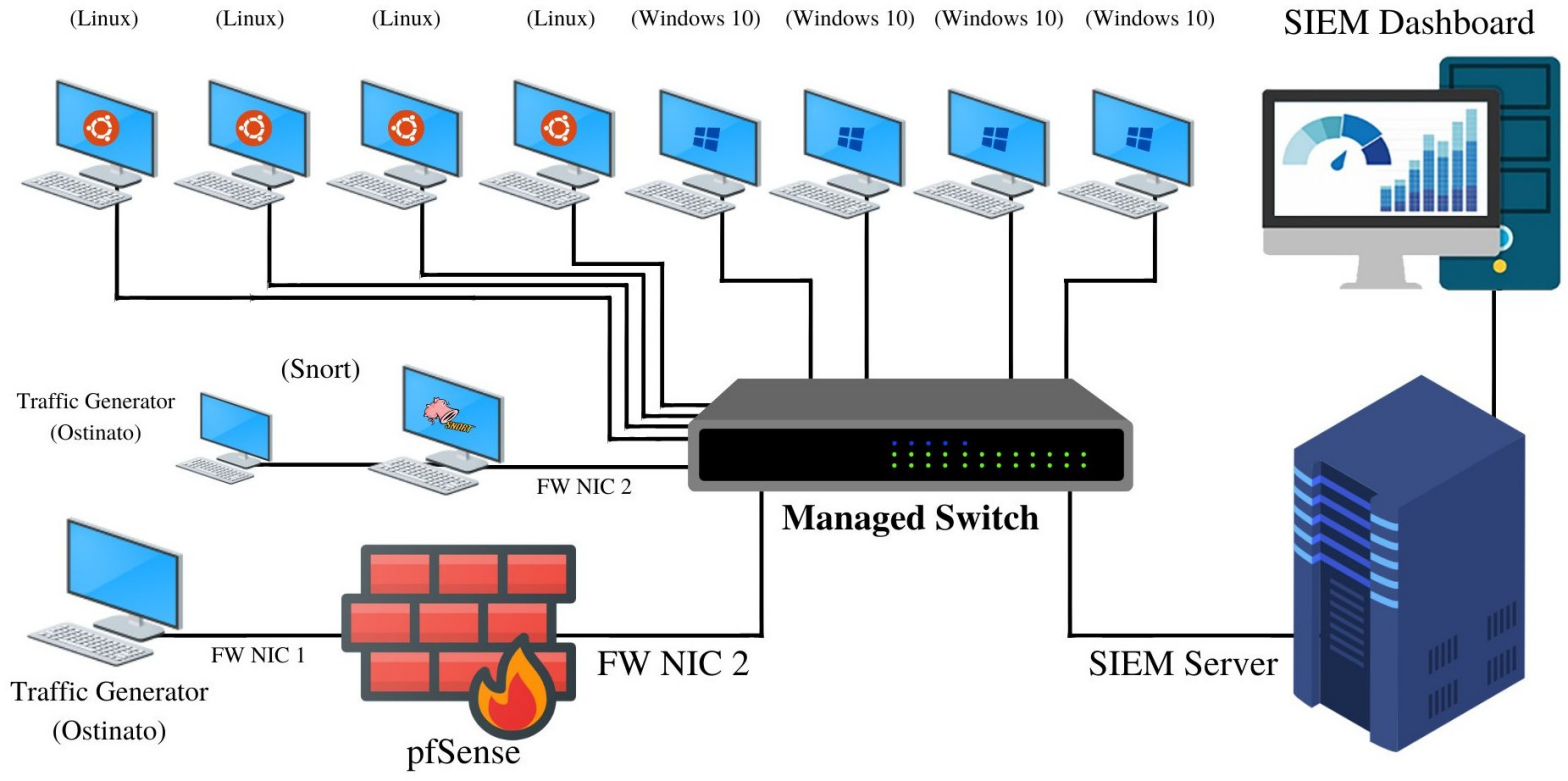
Test network for performance evaluation of SIEM.

To measure the maximum EPS capability of the SIEM solutions, we set up a test network that is representative of an enterprise network of SMEs (in terms of the variety of deployed devices and their configuration). The test network includes end-user devices that comprise a combination of Linux and Windows machines, pfSense firewall, Snort IDS and the four SIEM solutions namely Wazuh, SIEMonster, OSSIM, Elastic Security. The architecture of the test network is shown in Fig 2.

The event logs from end-user machines, IDS and firewall are directed towards the SIEM system via a mirror port configured on a managed switch. Hardware and software specifications of deployed devices are enlisted in Table 2.

**Table 2:** Hardware and software specifications for deployed infrastructure.

***CPU***	Intel Xeon Silver, Model 4214, 24 core
***OS***	ESXI 6.5.0 (Build 4564106) Win 10 Pro VMs Ubuntu VMs
***RAM***	128GB–2400 MHz
***Switch***	Cisco SG 350-52
***Software and Tools***	Ostinato v1.0 Python scripts for attack simulation
***Network Security Devices***	pfSense v2.6.0 Snort v2.9.2
***SIEM***	Wazuh v4.3.5 OSSIM v5.8.12 SIEMonster v4.6 Elastic Security v7.17.0

#### Simulation of DoS attack

One of the most common types of network attacks is the Denial-of-Service (DoS) attack that affects the availability of systems. In a DoS attack the adversary seeks to render a computer or another device inaccessible to its users by disrupting its regular operations. Typically, this is achieved by overwhelming a targeted machine with a large number of requests, to the point where it cannot process regular traffic. As a consequence, legitimate users are denied access to the service.

In order to simulate a DoS attack, we set up our testbed with traffic generators, IDS, Firewall and SIEM solutions, each running in a separate VM. We configure syslog in Snort and pfSense. The SIEM solution is configured to receive the logs. Next, we use Ostinato Traffic Generator to flood UDP traffic on WAN port of pfSense which blocks the incoming traffic. The firewall rules are triggered and the logs are forwarded to SIEM system through the forwarding port. Similarly, we flood traffic on WAN port of Snort which triggers IDS rules to generate logs for SIEM.

#### Simulation of malware attack

Malware is malicious software that can steal data or damage the systems. Malware comes in different forms such as viruses, worms, spyware, ransomware etc. We simulate a malware attack that makes changes to files on the end-user systems. There are three types of file changes that need to be monitored.

1. Create a new file—Spyware can add a malicious file that records the users’ keystrokes as they enter sensitive data such as passwords or credit card information.
2. Remove files—Worms can carry a malicious payload that removes critical files.
3. Modify files—Viruses can modify an existing file and try to hide their malicious code inside it.

In order to simulate such activities, we set up an experiment with multiple end-hosts that comprise Ubuntu and Windows machines. File Integrity Monitoring (FIM) systems on these machines examine the files for changes that may indicate an attack. They keep track of the files when they change, how they change, and who changed them by logging all the information. These logs are sent through agents that are deployed in the end-host systems. To enable FIM, we make the required changes in the configuration file of the SIEM agent. *Syscheck* is enabled to monitor the system checks in real time. The directories that need to be monitored are specified. We write a Python script to randomly create, modify and delete files in the monitored directory. As a result, the FIM module generates logs and forwards them to SIEM whenever a change is detected in the directory. This methodology is used for OSSIM, Wazuh, and SIEMonster.

In the case of Elastic Security, the Auditbeat agent is installed in the Ubuntu and Windows systems. Inside Auditbeat, the path for the directories that need to be monitored for changes is configured. Whenever changes are detected in these directories, logs are sent in real time to Elastic Security.

### 5.2 Performance results

**Fig 3:**
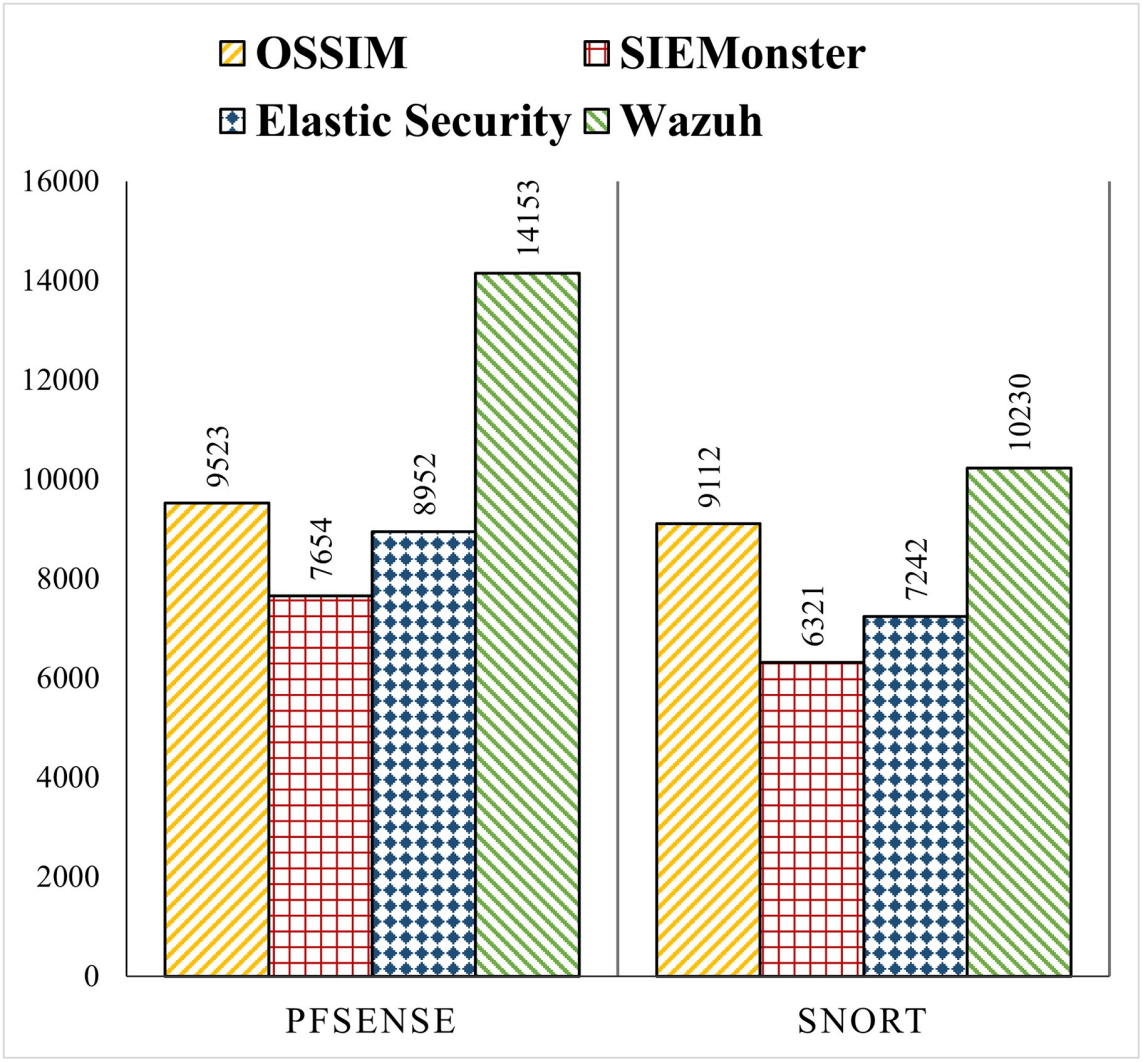
EPS comparison of SIEM systems with events of pfSense and Snort.

We perform experiments on all four SIEM solutions using the above-mentioned methodology. The default configuration without any customization is used in each SIEM solution to have a comparable baseline. Logs from Snort, pfSense, Windows and Linux machines are forwarded to the SIEM system and the EPS is measured. The performance comparison of the SIEM solutions with pfSense and Snort logs is shown in Fig 3. The x-axis shows pfSense and Snort, generating logs that are processed by the four SIEM systems while the y-axis shows the EPS achieved by each SIEM system.

As depicted in the graph, Wazuh has the highest EPS of around 14K, 10K for pfSense, Snort respectively. The next is OSSIM with EPS of 9.5K, 9.1K, 0.89K and 0.91K respectively. Elastic Security is in 3rd place with EPS of 8.9K, 7.2K, 0.95K and 0.96K respectively. The last is SIEMonster with EPS of 7.6K, 6.4K, 0.79K and 0.82K respectively. On average Wazuh has around 64% higher EPS than other SIEMs for pfSense, 38% higher for Snort, and more than 200% higher for Windows and Ubuntu.

We also note that pfSense events are processed faster as compared to Snort by all SIEM systems, despite the fact that both use Syslog to forward their events. The underlying reason is that Snort logs are more complex and they have a higher number of fields than pfSense, therefore more time is required to decode and process them. Similarly, the rule matching takes more time in the case of Snort as compared to the pfSense. Hence the EPS achieved in the case of pfSense is on average 20% more than Snort.

**Fig 4:**
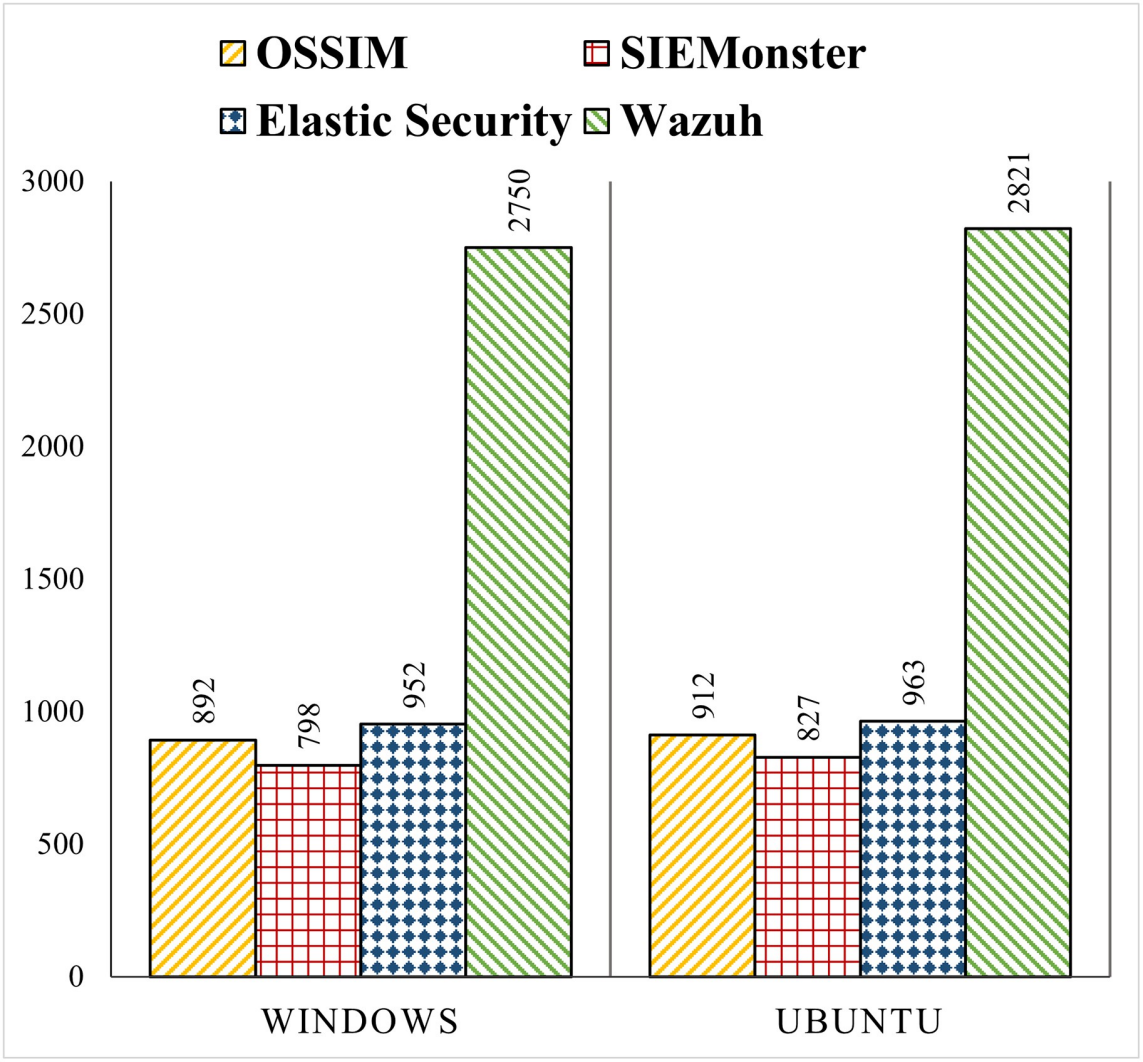
EPS comparison of SIEM systems with events of Windows and Ubuntu agents.

Fig 4 compares the EPS of the SIEM solutions using the events of FIM agent installed on Windows and Ubuntu at the end hosts. Again, Wazuh achieves the highest EPS of around 2.7K and 2.8K for Windows and Ubuntu respectively. The next is Elastic Security with EPS of 952 and 963, OSSIM 892 and 912, and SIEMonster 798 and 827 for Windows and Ubuntu respectively. The EPS of Wazuh is more than 200% higher than other SIEM solutions.

A comparison of Figs 3 and 4 also shows that the EPS achieved using the FIM agent is much lower compared to the Syslog used in pfSense and Snort. The reason is that the FIM logs received through the agent are larger in size and have more values. There are 25 fields in the FIM logs as compared to a maximum of 10 fields in Syslog for pfSense and Snort. In addition to the FIM logs, the agent also sends additional logs like rootkit detection, vulnerability scanning, and active response. These factors are responsible for slower processing and lower EPS in the case of the logs received through the agent.

### 5.3 Feature evaluation

Every SIEM solution has the basic capability to collect, store and correlate events from various devices in an organization’s IT infrastructure. Apart from this basic capability, various SIEM solutions can be differentiated and ranked based on a set of functional features. We have grouped the features into two sets namely, 1) Primary features and 2) Secondary features. As the name suggests, primary features are those that are essential for a SIEM system. Secondary features are desirable and add extra value to the system but are not crucial for a SIEM system.

#### Primary features

In order to ensure meaningful security evaluation of targeted SIEM solutions, it is important that all the essential features are considered. Thus we perform a detailed evaluation of these features for each SIEM solution and determine if the feature is missing, or has either a basic-level or advanced-level implementation.

1. **Event processing speed**
  One of the most important features for evaluating a SIEM system is its capability to detect events at high speed. The speed is measured in terms of EPS. We have experimentally evaluated and compared the EPS of the SIEM systems which have already been explained in detail in Section 5.
2. **Correlation Rules**
  The core purpose of a SIEM system is to detect events using correlation rules. Some SIEM solutions have rudimentary correlation rules while others have a more advanced and robust set of rules. We perform a quantitative as well as qualitative analysis of the correlation rules of each SIEM solution.
3. **Rule Customization**
  Every SIEM system comes with built-in correlation rules. At times, companies need to add custom rules tailored to their needs, so as to enhance the capabilities of the SIEM solution they are using. However, not all solutions allow customization. In this feature, we examine the rule customization capability of the targeted SIEM system.
4. **Log Integrity**
  The integrity of logs is very important for any SIEM system. If an adversary deletes legitimate events from the log while it is in transit from the agent to the SIEM server, correlation rules will not be fired and alarms will not be generated. In this feature, we evaluate the existence of mechanisms for ensuring the integrity of the logs sent by the agent to the SIEM server.
5. **Log Retention**
  SIEM solutions receive a large number of logs from a range of devices. Companies may be required to keep the logs in storage for several months for compliance purposes and forensic operations. This feature evaluates the duration for which the SIEM solutions retain the logs in their storage system.
6. **User Authentication**
  While user authentication is important for any system, the requirement for a secure and resilient authentication system for the SIEM solution is all the more essential due to the critical nature of the information available on the SIEM dashboard. This feature assesses the availability and the type of authentication mechanism.
7. **Access Control**
  Access control is an essential feature in a system to manage the roles and responsibilities of users. SIEM systems also require multiple roles such as Manager, Senior Analyst, and Junior Analyst and they have different levels of access to various modules of a SIEM system while they perform the day-to-day operations. In this feature, we evaluate the type of access control mechanism in the SIEM solutions.
8. **Fault tolerance**
  Fault tolerance is an important requirement for all critical systems. However, not every solution supports replication, high availability, disaster recovery, etc. SIEM systems are responsible for continuous monitoring and surveillance of end devices and network assets; therefore, it becomes important to examine the availability of fault tolerance capabilities of the SIEM solutions.
9. **Visualization**
  Data visualization is one of the most powerful features of a SIEM system. Lack of interactive exploration of data and customization of dashboards can adversely affect the effectiveness of a SIEM system.
10. **Compliance**
  Organizations often need to comply with multiple compliance regulations that provide guidelines and best practices based on the industry and type of data. These regulations help an organization in improving its information security strategy. Non-compliance with these regulations can result in a data breach and fines by the regulatory bodies. Some of the regulation and cybersecurity frameworks are explained next. The National Institute of Standards and Technology (NIST) is a voluntary framework that guides the management of cybersecurity-related risk by combining existing standards. It can be implemented by any organization. The International Organization for Standardization (ISO) provides various standards for security requirements related to the maintenance of information security management systems and risk management. The Health Insurance Portability and Accountability Act (HIPPA) is important for organizations that handle healthcare data such as hospitals, insurance companies, etc. The General Data Protection Act (GDPR) is important for businesses operating in Europe as it regulates the data protection of the citizens of the European Union. Payment Card Industry Data Security Standard (PCI-DSS) is a set of regulations for reducing credit card-related fraud and applies to companies handling credit card information. In this feature, we evaluate which compliance standards are satisfied by the studied SIEM systems.

#### Secondary features

Secondary features are defined as those features that can provide an added advantage but are not critical in the context of SMEs. For example, the built-in data sources provided by the SIEM solution may be enough for an SME. As the size of these organizations is not very large, a highly scalable solution is usually not a primary requirement. Vulnerability scanners are commonly available as standalone products and are already used by most SMEs, therefore the availability of vulnerability scanning capability as part of SIEM is not of primary importance. Similarly, threat intelligence is an advanced feature that is usually not considered by SMEs as they lack the financial and human resources to manage it. While active online community support is certainly beneficial, the documentation of the SIEM solutions is usually sufficient for configuration and troubleshooting. However, any SIEM solution that provides these secondary features will of course have an added advantage over others.

1. **Custom Data Sources**
  Most SIEM solutions provide the log parsers for commonly used devices by default. However, at times we might need to integrate a custom data source whose parser is not available in the SIEM system. Providing an easy-to-use mechanism for the user to write a plugin to parse the logs of custom devices can be very useful for the organization.
2. **Scalability**
  Organizations may grow in size with the passage of time and gradually add more sensors and devices. This feature considers the ability of a SIEM system to support the growing number of devices and events collected at the edge of the IT infrastructure.
3. **Vulnerability Scanning**
  Vulnerabilities on the networked systems can be exploited by attackers and may lead to information theft. Vulnerabilities may arise due to a number of reasons such as misconfiguration and deployments dependencies, etc. Thus, monitoring the network for vulnerabilities is important. This feature determines the availability of vulnerability scanning capabilities in the targeted SIEM.
4. **Threat Intelligence**
  Threat intelligence (TI) refers to the collection of data from multiple sources for further mining and devising a knowledge base for the threat landscape. From the perspective of SIEM, TI could be used for improving decision-making, policy management and rules augmentation. The availability of threat analysis tools using standard formats such as STIX, TAXII, JSON, etc. provides an added advantage to SIEM. This feature evaluates the level of threat intelligence support in a SIEM solution.
5. **Community and Support**
  Companies providing commercial products have a dedicated team that provides support and training to clients. Open-source projects thrive on community support. This feature evaluates the quality of documentation and active online community of developers and contributors of the SIEM solutions.

### 5.4 Scoring methodology

We have developed a methodology to assign an overall score to the target SIEM systems by using the primary and secondary features. Each feature has two components; the weight and the value. The primary features are absolutely necessary, while the secondary features are desirable but not mandatory. Therefore the weight of a primary feature is twice as much as a secondary feature, i.e., 2 for a primary feature and 1 for a secondary feature. The value of each feature is assigned based on the level of implementation of that feature, i.e., 0 if the feature is missing in a SIEM solution, 1 for basic implementation, and 2 for advanced implementation. The score of each feature is the product of its weight and value and the overall score of the SIEM system is the sum of the individual scores of all features. It is calculated by the following formula:

$$\begin{array}{c} \text{SIEMScore}=\sum_{i=1}^{n}W_{i}*V_{i} \end{array}$$ (1)

Here *i* is a primary or secondary feature, *V* is the value of the feature and *W* is the weight of the feature.

Suppose X is a primary feature and a SIEM system provides an advanced implementation of this feature then its score will be 2*3 = 6. Y is another primary feature with basic implementation, so its score will be 2*2 = 4. Z is a secondary feature that is not implemented, so its score will be 1*0 = 0. The total score of this particular SIEM solution will be 6+4+0 = 10. The maximum achievable score according to this methodology is 50. We evaluate the SIEM solutions and discuss their scores next.

## 6 Discussion

Security and performance evaluation of the four shortlisted SIEM systems is conducted as per the scoring methodology using 10 primary and 5 secondary features. The results are summarized in Table 3. The symbol × denotes that the feature is not available, ⋆ denotes basic implementation and ◇ denotes advanced implementation. Our study shows that Wazuh provides an advanced implementation of 13 features out of the 15 features we evaluated. Overall, Wazuh achieved the highest SIEM score of 47 followed by Elastic Security, SIEMonster and OSSIM with a score of 42, 37 and 26 respectively.

**Table 3:** Comparison of functional features of open source SIEM Solutions.

No.	Parameter	Score			
		Wazuh	OSSIM	SIEMonster	Elastic Security
	**Primary**				
1.	**EPS**	◊ 4	⋆ 2	◊ 4	⋆ 2
2.	**Correlation Rules**	◊ 4	⋆ 2	◊ 4	⋆ 2
3.	**Rule Customization**	◊ 4	◊ 4	× 0	◊ 4
4.	**Log Integrity**	◊ 4	⋆ 2	◊ 4	◊ 4
5.	**Log Retention**	◊ 4	⋆ 2	◊ 4	◊ 4
6.	**User Authentication**	⋆ 2	⋆ 2	◊ 4	⋆ 2
7.	**Access Control**	◊ 4	◊ 4	◊ 4	◊ 4
8.	**Fault Tolerance**	◊ 4	× 0	× 0	◊ 4
9.	**Visualization**	◊ 4	⋆ 2	◊ 4	◊ 4
10.	**Compliance**	◊ 4	× 0	◊ 4	◊ 4
	**Secondary**				
11.	**Custom Data Sources**	◊ 2	◊ 2	× 0	◊ 2
12.	**Scalability**	◊ 2	× 0	× 0	◊ 2
13.	**Vulnerability Scanning**	◊ 2	◊ 2	◊ 2	× 0
14.	**Threat Intelligence**	⋆ 1	⋆ 1	◊ 2	◊ 2
15.	**Community and Support**	◊ 2	⋆ 1	⋆ 1	◊ 2
	**SIEM Score**	47	26	37	42

***Symbols***:

◊ Advanced implementation ⋆ Basic implementation × Missing

### 6.1 Correlation rules

The comparison of the number of built-in correlation rules shows that Wazuh and SIEMonster have 3000+ rules while Elastic Security provides 300+ rules. OSSIM only has around 80 rules in the open source version while the commercial AlientVault USM version has 4500+ rules. If an organization wants to add custom rules for various use cases they can do so in all of the evaluated SIEMs except SIEMonster which does not provide any rule customization capabilities. OSSIM allows the users to add new correlation directives and cross-correlation rules via a Graphical User Interface (GUI). Similarly, Elastic Security also allows users to add new rules based on custom queries, thresholds, event correlation, or indicator matches. Custom query-based rule generates an alert when the rule’s query is matched. Threshold-based rule generates an alert when a specified field’s value appears a specified number of times in the log during a single execution. Event correlation rules need to be written using the Event Query Language (EQL) to match the results. Indicator match-based queries match the field values defined in the specified indicator index patterns and generate alerts accordingly.

### 6.2 Log integrity and retention

To ensure the integrity of logs, OSSIM uses AES-128 while the other three SIEMs use AES-256 encryption. Though AES-128 is considered secure, AES-256 is more desirable because it offers better protection due to the larger key length. When the logs are received by SIEM, they need to be stored for a long period. OSSIM retains logs for 5 days while the other SIEMs do not impose such limits and can store logs for a longer duration depending on the hardware storage capacity.

### 6.3 Authentication and access control

All SIEMs implement policies and procedures to ensure proper user identification management. In order to operate the SIEM system, the users need to be authenticated first using a username and password. The password policy usually requires a combination of uppercase letters, lowercase letters, numerical digits and special characters. SIEMonster additionally provides two-factor authentication which adds an extra layer of security. Similarly, all SIEMs implement role-based access control (RBAC), which provides the ability to allow administrative capabilities to certain users and restrict other users from accessing such capabilities. Wazuh provides two RBAC modes; allow list mode where everything is forbidden and the administrator needs to configure the roles to allow permissions, and a block list mode where everything is allowed and the administrator needs to restrict permissions for roles. OSSIM provides three RBAC roles, Read-Only, Analyst and Manager. Elastic Security allows assigning roles to users or groups and assigning privileges to various roles. SIEMonster also provides fine-grained role-based access control to indices, documents and fields.

### 6.4 Fault tolerance

Next, we evaluated another important feature of a SIEM system which is fault tolerance. High availability is usually provided by commercial SIEMs such as IBM QRadar using an arranged configuration between two cooperating appliances that use mirrored links to pass all traffic between them. This ensures that all of the traffic on the network is monitored by both the appliances and the state is maintained. In the case of the open source SIEMs, Wazuh and Elastic Security have a high availability deployment option that provides good fault tolerance. OSSIM and SIEMonster community edition does not provide high availability.

### 6.5 Visialuzation

SIEM systems collect a huge amount of data from diverse sources and proper data visualization methods are necessary for the analysis of security events. Wazuh, SIEMonster and Elastic Security use Kibana for dashboards and visualization which is extremely powerful. The users can create custom graphs and charts according to their requirements. OSSIM also provides data visualization but it is not as rich as the commercial USM version.

### 6.6 Compliance

Wazuh provides predefined compliance report templates for standards such as the Payment Card Industry Data Security Standard (PCI DSS), General Data Protection Regulation (GDPR), Health Insurance Portability and Accountability Act (HIPAA) and Good Practice Guide 13 (GPG 13). Elastic Security satisfies Federal Information Processing Standards (FIPS), Federal Information Security Management Act (FISMA), PCI DSS, HIPAA, GDPR, ISO standards. SIEMonster also provides compliance reports for NIST, HIPAA and other standards. OSSIM does not provide such reports.

### 6.7 Custom data sources

Support for adding new data sources is another important feature that allows the customization of a SIEM system. All SIEMs come with a set of built-in parsers and decoders for the logs of various devices. At times, the users may want to parse logs of a device that is not supported by SIEM by default. Wazuh allows users to add new decoders for the logs of their custom data sources. The decoder files are in XML format and use regular expressions for pattern matching. In Elastic Security data shippers known as Beats can be used to collect data from new sources. In OSSIM new plugins can be created for custom data sources. A unique plugin ID needs to be assigned and the path of the log file for the new data source needs to be configured. Regular expressions are used in the plugins to match the events. Finally, SIEMonster does not support customization.

### 6.8 Scalability

As the organizations grow in size, the requirements for event processing also increase. A Wazuh cluster which contains a group of cooperating managers can be set up for horizontal scalability. New nodes can easily be added to the cluster. Similarly, Elastic Security supports distributed deployment and new nodes can be added to the cluster to achieve scalability. The community edition of SIEMonster supports only a single server with up to 100 endpoints, while the multi-server and multi-tenant options are only available in paid versions. OSSIM is also limited in terms of scalability and does not support multi-tier distributed deployment.

### 6.9 Vulnerability scanning

Wazuh has the capability to detect vulnerabilities in the installed applications. Wazuh achieves this by building a global vulnerability database from Common Vulnerabilities and Exposures (CVE) repositories and generating alerts when a CVE affects a package installed on the monitored devices. It provides a full scan (scans every single package installed) and a partial scan (scans only new packages). OSSIM also provides a built-in vulnerability scanner to scan critical assets. The detected vulnerabilities can be used in cross-correlation rules and compliance reports. The scans can be scheduled as Immediate, Run Once, Daily, Day of the Week, Day of the Month, etc. SIEMonster provides vulnerability assessment along with other useful features such as penetration testing, Compliance checks, etc., using the PatrOwl platform. Elastic Security does not provide built-in vulnerability scanning capability.

### 6.10 Cyber threat intelligence

In terms of cyber threat intelligence, two of the evaluated SIEMs were found to have built-in threat intelligence capability. Elastic Security has a threat intelligence module that ingests threat data from various sources. This data is then utilized to detect malicious events via indicator match rules. This threat data is also used to add additional context to enrich the incoming traffic. SIEMonster provides threat intelligence using integration of Cortex and TheHive [55].

### 6.11 Community and support

Finally, we discuss the quality of documentation and a strong community where users can find answers to their questions and solutions to their problems regarding the configuration and customization of the SIEM solution. Wazuh, Elastic Security and OSSIM have very good documentation with detailed explanations of the working of various modules. Wazuh and Elastic Security have a strong community that provides valuable resources, forums and mailing lists for troubleshooting. Their community is active in the development and posts regular updates to ensure the solution remains up-to-date with security standards and new features.

In summary, the evaluation of these solutions indicates that Wazuh is the best open-source SIEM system for SMEs. It has the highest EPS in comparison with the other systems and it also scored the highest in the evaluation of important features. In addition, Wazuh provides the flexibility of customization as per the needs of the organization.

## 7 Conclusions and future work

SMEs face significant cybersecurity challenges in today’s digital landscape. A SIEM solution provides real-time monitoring and analysis of security events and helps in identifying potential threats and breaches promptly. It provides SMEs with a centralized view of their security posture, aggregating and correlating data from multiple sources and enables them to respond quickly to security incidents and mitigate potential damages.

This paper presents a technical analysis and evaluation of some of the most popular open-source SIEM solutions that are suitable for SMEs, namely, Wazuh, AlienVault OSSIM, SIEMonster and Elastic Security. In this work, we performed a detailed evaluation and comparison of the security and performance features of these SIEM solutions. For performance evaluation, we deployed each SIEM solution in a testbed that is representative of a typical enterprise network of an SME. EPS evaluation was done by generating logs from various network devices and collecting and processing them by the SIEM system. Feature evaluation was done through an in-depth analysis of the functional features of the SIEM systems. To the best of our knowledge, this is the first study that evaluates open-source SIEM solutions based on security and performance features by practically deploying and testing them in a testbed. Most of the prior work either provides a theoretical comparison of SIEM solutions or evaluates a single SIEM solution in detail.

We conclude that open-source SIEM solutions are essential for SMEs as they provide adequate security while staying within budgetary and resource constraints. However, not all open-source SIEM solutions provide the same level of security and performance. Our evaluation indicates that Wazuh is the best solution in terms of security features and performance. Wazuh provides plenty of features and should be the first choice for organizations looking for a customizable SIEM solution.

Although open-source SIEM solutions provide a powerful set of features that fit the requirements of most SMEs, they are not on par with their commercial counterparts. To improve the open-source SIEM solutions, we are currently working on some of the potential research directions. The first is the integration of unstructured Open Source Intelligence (OSINT) data by using Natural Language Processing (NLP) techniques. The second is the integration with Security Orchestration Automation and Response (SOAR) which will complement the capabilities of current SIEMs to create a proactive platform for early detection and response to various threats. The third research direction is to integrate Artificial Intelligence (AI) technologies in the core engines of open-source SIEMs in order to add predictive capabilities for user behavior analytics.
